# Effects of Semaglutide on Glycemic Control and Body Weight in Patients With Type 2 Diabetes: A Retrospective Cohort Study in a Primary Care Setting

**DOI:** 10.7759/cureus.82123

**Published:** 2025-04-12

**Authors:** Mahmoud Alzahrani, Lama Rammal, Razaz Felemban, Muhammed Khan, Hamza Saad, Muhammed Alrezqi, Nabil Alsulami, Abdulelah Alabdullatif, Zyad Alawashiz

**Affiliations:** 1 Family Medicine, King Saud Bin Abdulaziz University for Health Sciences College of Medicine, Jeddah, SAU; 2 Family Medicine, King Abdulaziz Medical City, Jeddah, SAU

**Keywords:** adverse effects, glycemic control, primary healthcare, real-world study, saudi arabia, semaglutide, type 2 diabetes, weight loss

## Abstract

Purpose: This study aimed to evaluate the effects of semaglutide (Ozempic) on glycemic control and body weight in patients with type 2 diabetes in a primary care setting. Additionally, the rate of discontinuation due to adverse effects was assessed.

Methods: We conducted a retrospective cohort study at a primary healthcare clinic in King Abdulaziz Medical City, Jeddah, Saudi Arabia. Data were extracted using BestCare, an interactive electronic health record system. The study included 238 adult patients with type 2 diabetes who initiated subcutaneous semaglutide at a starting dose of 0.25 mg and continued therapy for at least six months. Patients who were pregnant, lactating, or younger than 18 years were excluded. The primary outcomes were changes in glycated hemoglobin (HbA1c) and body weight.

Results: Among patients who adhered to the treatment regimen, maintained a healthy diet, and exercised regularly, significant reductions were observed in both HbA1c (from 8.2±1.54% to 6.9±1.44%; p=0.001) and weight (from 97 kg to 89.2 kg; p<0.001). However, 25.2% of patients discontinued semaglutide due to adverse effects.

Conclusion: Semaglutide is effective in improving glycemic control and promoting weight loss in patients with type 2 diabetes, particularly when combined with lifestyle modifications such as diet and exercise. Nonetheless, the potential for adverse effects may limit long-term adherence in a subset of patients.

## Introduction

Diabetes is a chronic metabolic disorder characterized by elevated blood glucose levels due to impaired insulin secretion or action. Glucose, the body's primary energy source, is regulated by insulin produced by the pancreas. Risk factors for diabetes include age, genetics, and obesity, all of which are also strongly associated with cardiovascular diseases such as coronary artery disease [1]. In a recent study conducted in Saudi Arabia, the prevalence of diabetes was reported at 23.7%, significantly higher than in previous national estimates. This increase was accompanied by a parallel rise in obesity, highlighting a growing public health concern [2].

Obesity and type 2 diabetes are closely intertwined. Weight loss is a key strategy in managing type 2 diabetes as it enhances glycemic control and reduces the risk of comorbidities. Individuals with both obesity and diabetes face greater vulnerability to cardiovascular complications. However, weight loss can be particularly challenging for these patients due to metabolic and hormonal alterations that favor weight gain. Moreover, many antihyperglycemic agents currently in use are associated with weight gain, further complicating diabetes management. Increased body weight is directly correlated with heightened insulin resistance [3].

Semaglutide is a long-acting, selective glucagon-like peptide-1 (GLP-1) receptor agonist approved for the treatment of type 2 diabetes and obesity. It mimics the effects of native GLP-1, reducing energy intake, increasing satiety, decreasing hunger, and improving glycemic control. Semaglutide also enhances glucose-dependent insulin secretion, suppresses glucagon release, and slows gastric emptying. It is available in both subcutaneous and oral formulations. Several GLP-1 receptor agonists, including semaglutide, have been approved in the United States and Europe, with others currently under review [4].

Clinical trials have demonstrated that semaglutide effectively improves glycemic control and facilitates weight loss in patients with type 2 diabetes. A 26-week study involving adults from diverse backgrounds reported significant reductions in both glycated hemoglobin (HbA1c) and body weight, with a low incidence of hypoglycemia and no fatalities [5]. Additionally, semaglutide has been shown to reduce the risk of cardiovascular events, including myocardial infarction and non-fatal stroke, when compared to placebo [6]. Long-term data also suggest that semaglutide may offer superior glucose and weight control compared to other GLP-1 receptor agonists, although further comparative studies are warranted [7].

Therefore, in this study, we aimed to evaluate the efficacy of semaglutide in glycemic control and weight reduction, as well as to determine the discontinuation rate due to adverse effects among patients with type 2 diabetes treated at a primary healthcare clinic in King Abdulaziz Medical City, Jeddah, Saudi Arabia.

## Materials and methods

Study design and setting

This retrospective cohort study was conducted at a primary healthcare clinic in King Abdulaziz Medical City, Jeddah, Saudi Arabia, after obtaining approval from the Standing Committee for Sabbatical Leaves, Publication and Research Ethics of the Ministry of Health, Saudi Arabia (approval number: 2024-5210). The study population included patients with type 2 diabetes who were prescribed subcutaneous semaglutide at an initial dose of 0.25 mg.

Participants

Eligible participants were adults aged ≥18 years who began semaglutide treatment between March 2021 and March 2022. Patients who were pregnant, lactating, or younger than 18 years were excluded. All eligible patients during this period, as approved by the Specialized Polyclinic-Primary Health Care, were considered for inclusion.

A total of 279 patients received semaglutide during the study period. A minimum sample size of 162 was calculated (95% confidence level, 50% response distribution, 5% margin of error). Consecutive sampling was employed to maximize representativeness in the relatively small eligible population. After applying the inclusion criteria, 238 patients were included in the analysis.

Data collection

Data were collected between June and September 2022 by five trained reviewers using BestCare, an interactive electronic health record system. Collected variables included demographic data (age and sex), clinical measures (weight, body mass index (BMI), HbA1c, blood pressure, fasting blood sugar, and albumin-to-creatinine ratio (ACR)), and lipid profile (total cholesterol, high-density lipoprotein (HDL), low-density lipoprotein (LDL), and triglycerides before and after treatment). Medication history was also reviewed, including concomitant use of antidiabetic agents such as gliclazide, metformin, pioglitazone, sitagliptin, dapagliflozin, insulin, and prior liraglutide use. Lifestyle factors were assessed, including dietary adherence and physical activity. Clinical events were documented, including hypoglycemic episodes and diabetes-related complications (cardiovascular disease, neuropathy, nephropathy, and retinopathy).

Compliance was defined as semaglutide use for ≥6 months. Discontinuation due to adverse effects was recorded. When necessary, telephone follow-up was conducted to clarify missing data, confirm compliance, or verify reasons for discontinuation. Verbal informed consent was obtained during these calls to ensure patient privacy and data integrity.

Outcomes

The primary outcomes were changes in HbA1c and body weight after at least six months of semaglutide use. Secondary outcomes included rates of treatment discontinuation due to adverse effects and associations with lifestyle factors.

Statistical analysis

Numerical variables were analyzed using parametric or non-parametric methods, as appropriate. Categorical variables were expressed as frequencies and percentages. The chi-squared test or Fisher's exact test was used for group comparisons. Statistical significance was set at p<0.05. Data analysis was performed using IBM SPSS Statistics for Windows, Version 20.0 (Released 2011; IBM Corp., Armonk, New York, United States).

## Results

A total of 238 patients were included in the analysis. The mean age of the cohort was 54.5±10.08 years, with 154 (64.7%) male and 84 (35.3%) female patients (Table 1).

**Table 1:** Demographic and clinical characteristics of the study group (N=238) Data are presented as mean±standard deviation for continuous variables and as percentages for categorical variables. For continuous variables, median and IQR are also reported where appropriate. BMI: body mass index; HbA1c: glycated hemoglobin; IQR: interquartile range

Characteristics	Study group (N=238)
Age, years	54.5±10.08
Female sex	84 (35.7%)
Male sex	154 (64.7%)
BMI, kg/m²	35.29 (31.58-40.03)
HbA1c, %	8.7±1.84
Weight, kg	92.3±18.84

Impact of semaglutide on weight

Semaglutide usage significantly reduced body weight (p<0.001). In compliant patients, the mean weight decreased from 93.5±17.70 kg before treatment to 88.8±18.47 kg after treatment. A paired t-test demonstrated a significant weight reduction in patients who were compliant with semaglutide, diet, and exercise. The mean weight change was from 97±17.97 kg to 89.2±15.28 kg (p<0.001).

Impact of semaglutide on glycemic control

A significant reduction in HbA1c was observed in compliant patients who followed a diet and exercise regimen. The mean HbA1c decreased from 8.2±1.54% to 6.9±1.44% (p<0.001). In patients who were compliant but did not follow the recommended lifestyle changes, the mean HbA1c reduced from 8.7±1.77% to 7.6±1.76% (p<0.001). A paired t-test showed a significant association between semaglutide use for ≥6 months and the reduction in HbA1c (p<0.001).

Treatment discontinuation due to adverse effects

Sixty patients (25.2%) did not complete the recommended 1 mg weekly dose of semaglutide. Adverse effects were the primary reason for discontinuation. Of the 60 patients, 28 (46.7%) experienced nausea and vomiting, 12 (20%) had abdominal pain, 10 (16.7%) reported headaches, seven (11.7%) discontinued due to medication stockouts, and two (3.3%) reported constipation. Five patients (8.7%) discontinued after achieving their weight loss target.

Laboratory data

Regarding kidney function, the median ACR was 1.8 (interquartile range (IQR): 1-6.4) before semaglutide treatment and remained the same after treatment (1.8; IQR: 0.9-6). A paired t-test showed a significant decrease in ACR levels (p=0.039). Additionally, total cholesterol and LDL cholesterol levels significantly decreased. The mean total cholesterol level decreased from 4.6±0.90 mmol/L to 4.4±0.97 mmol/L (p=0.004), and LDL cholesterol decreased from 2.5±0.85 mmol/L to 2.3±0.86 mmol/L (p=0.025).

## Discussion

This study evaluated the effects of once-weekly subcutaneous semaglutide treatment in patients with obesity and type 2 diabetes. We specifically examined patients who started at the recommended 0.25 mg dose, which allowed us to better understand their medical history and account for external variables that might affect outcomes.

Unlike a study that focused on weight loss effectiveness among obese or overweight patients without diabetes [8,9], our study concentrated on those with type 2 diabetes. In diabetic patients, increased body weight is typically secondary to insulin resistance, a condition that can be managed effectively with a GLP-1 receptor agonist such as semaglutide. In contrast, obese or overweight patients without diabetes may experience weight gain due to thyroid abnormalities, neurological disorders, stress, depression, and other factors, making it crucial to address underlying causes first. Despite differences in population selection, our findings of an 8.1% weight reduction are comparable to those of previous studies on obese and overweight patients, which reported a 7.9% reduction [10].

In a study of semaglutide use in obese patients, where the dose was gradually increased to 2.4 mg, a 14.9% weight reduction was observed, greater than the 8.1% reduction in our study, where the maximum dose was 1 mg [10]. This difference highlights the potential for increased weight loss with higher doses of semaglutide, though further studies are needed to confirm this finding.

Our study also found that the combination of subcutaneous semaglutide use and lifestyle modifications led to a significant reduction in HbA1c levels, suggesting improved glycemic control. The reduction in HbA1c from 8.7% to 6.9% in patients who were compliant with semaglutide, diet, and exercise aligns with findings from previous studies demonstrating the efficacy of semaglutide in managing blood sugar [11].

However, despite starting at a low dose of 0.25 mg, 25.2% of patients discontinued treatment due to adverse effects, with the most common being gastrointestinal disturbances such as nausea, vomiting, abdominal pain, and headache. These findings are consistent with previous studies on semaglutide and other GLP-1 receptor agonists, which have reported similar adverse effect profiles [12-14].

Our results suggest that semaglutide, starting at 0.25 mg and gradually increasing to 1 mg, could be a promising treatment option for patients with type 2 diabetes, with a reduced risk of severe adverse effects. The best outcomes were seen in patients who incorporated lifestyle modifications, highlighting the importance of diet and exercise in diabetes management. Future studies should consider incorporating these factors to account for variability in patient responses.

This study provides valuable insight into the use of semaglutide in primary healthcare settings in Saudi Arabia, making it one of the first to evaluate the effects of this once-weekly GLP-1 receptor agonist on weight and glycemic control in this population. The strength of this study lies in the high follow-up rate after six months, though a longer follow-up period would have been beneficial to fully assess long-term efficacy and adverse effects.

A limitation of this study is its relatively short duration and small sample size due to the novelty of semaglutide. Future research could expand on these results by using a larger sample, a randomized clinical trial design, and a longer follow-up period to provide a more comprehensive understanding of semaglutide's effects in diverse clinical settings.

## Conclusions

Weekly semaglutide treatment in patients with type 2 diabetes resulted in significant improvements in both glycemic control and weight management, with the most notable weight reduction observed in patients who were more consistent with diet, exercise, or both. Additionally, semaglutide use contributed to general improvements in related laboratory markers. While most patients continued treatment until reaching the highest recommended dose of 1 mg, 60 patients discontinued due to adverse effects. These findings highlight the potential of semaglutide as an effective therapeutic option for diabetes management, though attention to adverse effects is necessary for optimal patient adherence.
